# Antioxidants in Arrhythmia Treatment—Still a Controversy? A Review of Selected Clinical and Laboratory Research

**DOI:** 10.3390/antiox11061109

**Published:** 2022-06-02

**Authors:** Jakub Szyller, Dariusz Jagielski, Iwona Bil-Lula

**Affiliations:** 1 Department of Medical Laboratory Diagnostics, Division of Clinical Chemistry and Laboratory Haematology, Faculty of Pharmacy, Wroclaw Medical University, 50-556 Wroclaw, Poland; iwona.bil-lula@umw.edu.pl; 2 Department of Cardiology, Centre for Heart Diseases, 4th Military Hospital, 50-981 Wroclaw, Poland; dariuszjagielski@gmail.com

**Keywords:** antioxidants, atrial fibrillation, ventricular arrhythmia, vitamin C, vitamin E, resveratrol

## Abstract

Antioxidants are substances that can prevent damage to cells caused by free radicals. Production of reactive oxygen species and the presence of oxidative stress play an important role in cardiac arrhythmias. Currently used antiarrhythmic drugs have many side effects. The research on animals and humans using antioxidants (such as vitamins C and E, resveratrol and synthetic substances) yields many interesting but inconclusive results. Natural antioxidants, such as vitamins C and E, can reduce the recurrence of atrial fibrillation (AF) after successful electrical cardioversion and protect against AF after cardiac surgery, but do not affect the incidence of atrial arrhythmias in critically ill patients with trauma. Vitamins C and E may also effectively treat ventricular tachycardia, ventricular fibrillation and long QT-related arrhythmias. Another natural antioxidant—resveratrol—may effectively treat AF and ventricular arrhythmias caused by ischaemia–reperfusion injury. It reduces the mortality associated with life-threatening ventricular arrhythmias and can be used to prevent myocardial remodelling. Statins also show antioxidant activity. Their action is related to the reduction of oxidative stress and anti-inflammatory effect. Therefore, statins can reduce the post-operative risk of AF and may be useful in lowering its recurrence rate after successful cardioversion. Promising results also apply to polyphenols, nitric oxide synthase inhibitors and MitoTEMPO. Although few clinical trials have been conducted, the use of antioxidants in treating arrhythmias is an interesting prospect.

## 1. Introduction

Oxidative stress (OS) is involved in the pathogenesis of cardiovascular diseases, such as hypertension, heart failure, atherosclerosis and ischaemia/reperfusion injury (IRI) [1,2,3,4,5,6]. Reactive oxygen species (ROS) production is also associated with cardiac arrhythmias [7,8,9]. So far, scientific studies have mainly focused on the role of OS in atrial fibrillation (AF). Little is known about the role of ROS and OS in the development of ventricular fibrillation (VF), ventricular tachycardia (VT) or other ventricular arrhythmias (VAs). VT and VF are the most common life-threatening arrhythmias leading to sudden cardiac arrest [10]. The use of antioxidants could contribute to better treatment of cardiac arrhythmias. A significant group of patients are people with heart failure (HF) and left ventricle ejection fraction (LVEF) <35% with implanted cardiac resynchronisation therapy devices with defibrillators (CRT-D) or implantable cardioverter-defibrillators (ICDs). ICDs are used for primary and secondary prevention in patients with HF. In these patients, OS is a critical pathophysiological pathway in the development and progression of HF [3,11] and possibly arrhythmias. The exact role of OS in arrhythmia development in these patients is complicated, since the intervention of the ICD also influences ROS production. Electric shocks can cause cellular myocardium damage and abnormalities in OS and calcium regulation, and can stimulate pro-inflammatory factors, which can paradoxically promote reinduction of VAs [12]. This paper presents an overview of the current pathophysiology of ROS/oxidative stress-related cardiac arrhythmias and a review of studies using the most commonly tested antioxidants in humans and animals to prevent arrhythmias. Data on supraventricular (AF) and ventricular arrhythmias (VT, VF) are presented. Understanding the role of OS in arrhythmia induction will help improve treatment methods and reduce the risk of complications.

## 2. Main ROS Molecular Mechanisms Inducing Arrhythmias

**Figure 1:**
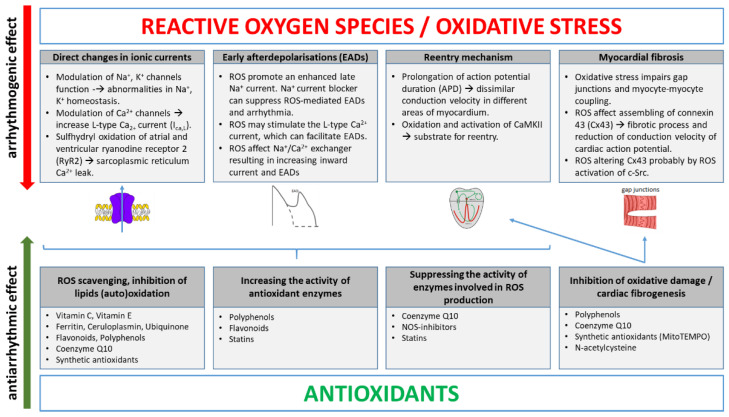
Main molecular mechanisms of ROS and selected antioxidants in cardiac arrhythmias.

There are several possible ways for ROS to induce arrhythmias (Figure 1). One includes increasing focal activity, where via an early afterdepolarisation (EAD) mechanism [13,14], ROS can modify substrate for re-entry [15] and affect several ionic currents in cardiomyocytes [16]. One exciting study indicated that perfusion of the heart with H_2_O_2_ in the Langendorff animal model induces VT/VF and AF, providing solid and compelling evidence that ROS elevation can cause arrhythmia [13]. Oxidative stress may have a significant arrhythmogenic potential for inducing AF by Ca2+ releasing and enhancing inadequate electrical activity [17], but the mechanisms linking the redox state to AF are not well understood. ROS may alter Ca^2+^ homeostasis by inhibiting the Ca^2+^-ATPase pump and activating the Ca^2+^ release channel [18]. One of the cellular oxidants—H_2_O_2_—seems to work as an oxidant of key thiol groups, which may be one of the molecular mechanisms involved in the action of ROS and atrial remodelling [18]. In atrial myocytes, type 2 ryanodine receptor (RyR2) is the major intracellular Ca^2+^ release channel, and its oxidation may be associated with the occurrence of AF [19]. This study showed that chronic AF patients had atrial RyR2s, which were oxidized, phosphorylated and depleted of calstabin-2 [19]. It leads to increased sarcoplasmic reticulum Ca^2+^ leak and the development of AF. Decreased ratios of reduced to oxidized glutathione (GSH/GSSG) and reduced to oxidized cysteine were also found in the blood samples of AF patients [20]. Atrial biopsies from 19 patients (mainly men, mean age of 49 years) with persistent AF showed increased levels of heme oxygenase-1 (HO-1) and 3-nitrotyrosine (3-NT) in atrial tissue [21]. Several AF-associated conditions cause NADPH oxidase activation, a major contributor to atrial OS. Combined with nitric oxide synthase (NOS) dysfunction, this leads to superoxide formation in atrial myocytes and the NADPH activity is independently associated with AF after cardiac surgery [22,23]. Magnesium (Mg) also plays an important role. It has been shown to reduce arrhythmias, such as AF [24], torsade de pointes (TdP) [25] and other VAs [26]. Mg deficiency may cause OS. Decreased mitochondrial Mg levels may explain the mitochondrial ROS overproduction and decreased ATP levels [27]. The influence of magnesium on the development of OS is very complicated, as it concerns the suppression of the antioxidant system, amelioration of mitochondrial calcium overload and the function of mitochondrial ATP-sensitive potassium channel, among other factors [28]. It results in depolarisation of the mitochondrial membrane potential. OS also increases VAs by reducing sodium channel currents and preventing its complete inactivation, resulting from lipid peroxidation [29]. The relationship between arrhythmia and oxidative stress also arises from the concentration of urinary 8-hydroxy-2′-deoxyguanosine (8-OHdG) and VT [30]. Even the most recent studies (2021) in elderly patients after coronary stent implantations seem to unquestionably confirm the key role of OS in cardiac arrhythmias [31]. ROS and oxidative heart damage are especially important in the development of myocardial remodelling and HF, a serious medical problem worldwide. ROS production in HF is associated with chronic neurohormonal activation and the consequent upregulation of angiotensin II and increased stress factors, such as pressure overload or hypoxia [32]. The primary sources of ROS in the heart are the mitochondrial electron transport chain (which produces, under the pathological conditions, large quantities of superoxide), xanthine oxidase, NADPH oxidases and uncoupled nitric oxide synthase (NOS). Pathological stimuli, such as mechanical stretch or pro-inflammatory cytokines, cause an increased expression and activity of xanthine and NADPH oxidase, leading to increased production of ROS, cellular dysfunction and injury. ROS also stimulate cardiac fibroblast proliferation and myocardial fibrosis, and affect gap junction channels participating in intercellular communication and action potential propagation [14]. They activate the matrix metalloproteinases (MMPs), leading to extracellular matrix remodelling. Cardiac connexin-43 (Cx-43) and pannexins, which are a component of gap junction, have been the subject of research [33]. These changes cause hypertrophy, fibrosis, apoptosis and heart muscle contractile dysfunction. Moreover, there is a significant decrease in the activities of antioxidant enzymes [34]. Direct myocytic electrical aberrations due to ROS and myocardial remodelling can contribute to arrhythmias. These changes can cause arrhythmias not only in HF, but also in other conditions, with increased OS described in several cardiovascular diseases, including hypertension, dyslipidaemia, peripheral artery disease or metabolic syndrome. Detailed descriptions of molecular mechanisms can be found in recently published papers [28,35].

## 3. Antioxidants in Arrhythmia Treatment

### 3.1. Vitamins C and E in Atrial Fibrillation and Other Supraventricular Arrhythmias

#### 3.1.1. Animal Studies

The influence of antioxidants on developing arrhythmias was noted a long time ago. In 1987, Frolkis et al. showed that α-tocopherol inhibited the arrhythmogenic effects of CaCl2, vasopressin and epinephrine in rats [36]. It was found that the administration of 50 mg/100 g i.p. vitamin E for five days limits the development of arrhythmias. Animals treated with tocopherol had significantly lower incidence rate of sinus arrhythmia (16.6% vs. 75% in control group), wandering pacemaker (16.6% vs. 58.3%), ventricular extrasystoles (41.6% vs. 100%) and paroxysmal tachycardia (0% vs. 33.3%) [36]. Another experiment showed that rats in the tocopherol group with epinephrine-induced arrhythmia had significantly lower glutathione reductase activity and higher total content of SH-groups [36]. Therefore, vitamin E exerts important antiarrhythmic, antioxidant and cardioprotective effects by inhibiting epinephrine-induced lipid peroxidation. This observation may be of particular importance in patients treated in intensive care units with high doses of catecholamines, or in patients with catecholaminergic polymorphic ventricular tachycardia (CPVT).

#### 3.1.2. Human Studies

It was also noted a long time ago that the administration of vitamin C before and after successful electrical cardioversion of AF reduces early recurrence rates from 36.3% in the control group to only 4.5% in patients who received vitamin C [37]. It is worth paying more attention to antioxidants before planned cardioversion, or in patients with implanted cardiac resynchronisation therapy defibrillators (CRT-D) or ICDs, in order to increase treatment effectiveness. Currently, there is a significant amount of research into the use of antioxidants in AF; however, the results are inconclusive [38,39,40,41,42]. There are at least several pathophysiological mechanisms, and each of them requires further research. Interesting results were found in 203 patients with post-operative AF (POAF) in a randomised, double-blind placebo-controlled trial [38]. Two days before cardiac surgery, the patients were supplemented with 1 g/day of vitamin C, 400 IU/day of vitamin E and 2 g/day of n − 3 polyunsaturated fatty acids (PUFAs) immediately after randomisation. As a result, 32% of patients in the placebo group and only 9.7% in the treatment group developed AF. The placebo group had a 3.62 times higher risk of POAF on any day by comparison with supplemented patients. They also had higher activity of antioxidant enzymes and enzyme gene-expression levels (catalase (CAT), superoxide dismutase (SOD) and glutathione peroxidase (GPx)) in the atrial tissue. Overall, a 66% reduction in atrial tissue susceptibility to arrhythmia was demonstrated [38]. A very simple and safe method provided a significant risk reduction of POAF by over 20%, by increasing the activity of antioxidant enzymes in the heart. POAF complicates up to 20%–40% of cardiac surgical procedures, and 10%–20% of non-cardiac thoracic operations, presenting a serious clinical problem. Antioxidants can reduce OS in inflammation or cardiac ischaemia after surgery, which is one of the POAF development mechanisms. Mirhoseini et al. presented antioxidant supplementation and atrial arrhythmias in critically ill trauma patients, representing a very diverse group [41]. AF is the most common arrhythmia and is independently associated with higher mortality in trauma patients [43]. One of the studies used a high dose of an antioxidant (vitamin C—1000 mg orally, per tube or intravenously every 8 h, vitamin E—1000 units orally or per tube every 8 h and selenium—200 mcg orally, per tube or intravenously, daily for 7 days in total) during a two-week hospitalisation or before discharge, and showed the differences between the antioxidant and control groups. No differences were found in the hospital mortality rates, but the use of antioxidants was associated with a longer expected survival time [41]. Antioxidants may have antiarrhythmic effects, but they also reduce the inflammatory reaction severity, an important arrhythmogenic factor often found in injuries and polytrauma. Vitamin C decreases the spontaneous activity of the pulmonary vein and attenuates the arrhythmogenic effects of H_2_O_2_ in rabbits due to the intensification of ectopic beats [17]. This effect is related to the increase in Ca^2+^ release and can be important when performing ablation procedures. The meta-analysis of 15 clinical trials with 1738 subjects indicated that antioxidants, such as ascorbic acid, α-tocopherol, N-acetylcysteine and a mix of omega-3 polyunsaturated fatty acid (PUFA) + ascorbic acid + tocopherol, reduced POAF (21% vs. 34% in the control group) incidence after coronary artery bypass grafting (CABG) or valve replacement [44]. A meta-analysis of 11 randomised controlled trials with 3137 patients revealed that the use of n-3 PUFA without antioxidants did not reduce the incidence of POAF compared with the control group. However, the combination therapy with vitamins C and E decreased the incidence of POAF by 68% [45].

### 3.2. Vitamins C and E in Ventricular Arrhythmias

#### 3.2.1. Animal Studies

Ventricular arrhythmias are a serious medical problem and can be life-threatening. Sethi et al. indicated that rats with epinephrine-induced arrhythmia treated with vitamin E (20 mg/kg/day for 21 days) had a significantly lower VT incidence (by approx. 50%), nonsustained VTs and arrhythmia duration. Simultaneously, the malondialdehyde (MDA) level was lower than in the control group [46]. Vitamin E (25 mg/kg/day) also led to decreased premature ventricular contractions (PVCs) in rat hearts 21 days after coronary artery occlusion [47]. An ischaemia–reperfusion experiment with sheep indicated that vitamin C and deferoxamine (1.5 g ascorbic acid and 1g deferoxamine with 500 mL NaCl 0.9%) could protect the myocardium against VAs. Ischaemia was caused by ligating the left anterior descending coronary artery for 45 min. In the control group, 100% of animals in the reperfusion phase had VT/VF. In the vitamin C + deferoxamine group, only 37.5% of animals presented VT/VF [48]. Ischaemia/reperfusion injury and OS also induce prolongation of QT interval and action potential duration, which may result in polymorphic ventricular tachycardia. Chen et al. indicated that QT prolongation is associated with ROS-related inhibition of delayed rectifier potassium channel (IKr). In a guinea pig heart model, treatment with vitamin E shortened the QTc interval from 296.9 ms during I/R to 268.1 ms after tocopherol treatment, and the QT interval progressively increased at the time of vitamin E administration [49]. It shows an important role of antioxidants in treating QT interval-related arrhythmias. It appears that antioxidants may improve the treatment outcomes in patients with ischaemic heart disease or myocardial ischaemia reperfusion syndrome. Reperfusion strategies are the current standard therapy for acute myocardial infarction (AMI) and are associated with the production of high levels of ROS. Ventricular arrhythmias generated in the reperfusion phase may be life-threatening, and their treatment with standard antiarrhythmic drugs is also not entirely safe. Vitamins C and E may improve safety in patients with AMI or congenital or acquired long QT syndrome. The use of antioxidants or ROS-scavengers with drugs that prolong QT may be a promising therapeutic option in reducing the risk of side effects.

#### 3.2.2. Human Studies

Interesting conclusions have been reached concerning patients with chronic Chagas disease, who have a higher prevalence of PVCs caused by increased oxidative stress [50]. Forty-one patients received antioxidants, such as vitamins C (500 mg/day) and E (800 UI/day), for six months. A 24-hour Holter monitoring device was used to detect arrhythmias. The prevalence of PVCs (incidents) in patients with advanced heart disease was 5391 before antioxidant supplementation vs. 1185 after supplementation. The reduction in PVCs was accompanied by a decrease in serum markers of oxidative stress. The activities of SOD, GPx, and glutathione reductase were decreased in all patients in whom vitamins C and E were used [50]. Rarely, frequent PVCs can lead to chaotic, dangerous heart rhythms and possibly sudden cardiac death, and therefore require treatment. Therefore, a new and safe therapy for reducing oxidative stress is highly desirable. It appears that antioxidant therapy could benefit the treatment of sudden cardiac arrest in the VT/VF mechanism.

**Figure 2:**
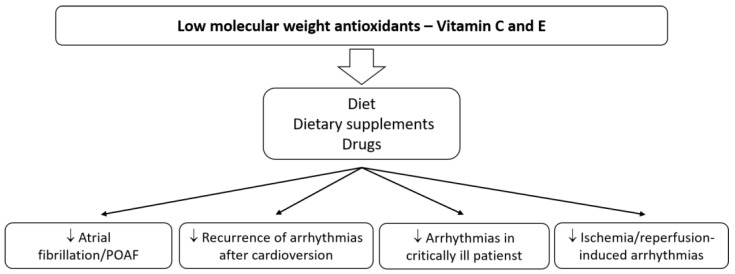
The main effects of vitamins C and E on cardiac arrhythmias.

The above data from studies in animal models and patients of the cardiology departments indicate that the use of natural antioxidants, e.g., vitamin C or E, may benefit patients with supraventricular and ventricular arrhythmias (Figure 2). Using antioxidants contained among other nutrients in food, could be a simple and safe method of preventing or reducing the risk of developing, for instance, the most commonly treated arrhythmia (AF) in patients after cardiac surgery or of the recurrence of arrhythmias after cardioversion. Post-operative arrhythmias represent a significant source of morbidity and mortality. Better therapies are essential for patients taking antiarrhythmic drugs, which show only moderate antiarrhythmic efficacy, with a concurrent risk of proarrhythmia and adverse organ effects. Patients on a PUFA-rich diet can also benefit.

### 3.3. Resveratrol and Atrial Fibrillation

Fascinating research has been conducted concerning the application of resveratrol (RSV) in arrhythmia treatment. RSV (3,5,4′-trihydroxy-trans-stilbene) is a polyphenolic compound present in a variety of plant species and in wine. It is a scavenger of a number of free radicals and is thought to have cardiovascular benefits [51]. As a polyphenolic compound, it works as a free radical scavenger, but many of its protective effects in vivo are mediated by gene regulations [52]. It can influence the expression of nearly 800 genes and participate in regulating over 140 signalling pathways [53]. Unfortunately, we did not discover many studies investigating the antiarrhythmic effect of resveratrol in humans. All current experiments are based mainly on animal models. Much of the human-based research has focused on metabolic effects [54].

#### 3.3.1. Animal Studies

As AF is the most common arrhythmia, much research has focused on the use of antioxidants in AF. A recent study showed that RSV reduces AF susceptibility in the heart via the PI3K/Akt/eNOS signalling pathway [55]. It indicated that RSV decreased atrial fibrosis and regulated ion channel function to reduce AF in rabbits with HF. AF episodes were significantly reduced in the RSV-administered group compared with the control group (5.0% vs. 38.0%). Moreover, the treatment reduced the incidence of triggered activities [55]. The inducibility and duration of AF in a collagen-induced arthritis animal model was able to be significantly reduced by RSV [56]. RSV inhibited atrial myocyte apoptosis and fibrosis. This indicates the potential use of this compound to prevent myocardial remodelling. This process is related to OS. RSV can also reduce cardiac arrhythmia; that is, prevent premature atrial contraction affecting the ion channels [57]. Antiarrhythmic actions of RSV may be mediated through INa, Ito, Iss and Ca^2+^channel blockade [58]. Similarly, by inhibiting the ionic channels, RSV could contribute to protection against ischaemia/reperfusion-induced lethal arrhythmias. RSV induced a prolongation of the atrial effective refractory period and slowed conduction velocity, which is a critical antiarrhythmic mechanism. A study on rabbit hearts demonstrated that RSV and piceatannol (a derivative of RSV) reduce AF incidence after administration of acetylcholine and isoproterenol [59]. Thus, RSV and vitamins C and E can improve the outcomes of patients with supraventricular and ventricular arrhythmias, including those in a critical condition, with risk factors and with diseases resulting in myocardial remodelling and structural changes.

#### 3.3.2. Human Studies

Conversely, human studies looking at wine (alcohol) consumption as a source of RSV produce conflicting results. A large cohort study of 79,019 Swedish men and women indicated that consuming >14 drinks per week of wine was associated with an increased risk of AF [60]. These findings indirectly call into question theories on the antiarrhythmic effects of resveratrol in humans. However, it should be remembered that the conflicting studies were designed differently, and it is often impossible to unequivocally compare the doses of alcohol and/or RSV consumed. The effect of RVS may be different when used alone, e.g., as an extract, from when it is present in an alcoholic drink. More clinical trials of RSV supplementation are needed, but the initial results are promising. Grapes, especially their skins, and blackberries and blackcurrants are the main RSV sources. The use of RSV and/or the Mediterranean diet as supportive therapy may positively influence the outcome of AF treatment.

### 3.4. Resveratrol and Ventricular Arrhythmias

#### Animal Studies

Beneficial antiarrhythmic effects of RSV were demonstrated more than 20 years ago in animal studies, among others. Male Sprague–Dawley rats (200–300 g) underwent myocardial ischaemia by occlusion of the left descending coronary artery and were infused with a bolus of resveratrol’s different doses 15 min before coronary occlusion [61]. Pretreatment with RSV did not affect ischaemia-induced arrhythmias (100% of the rats had VT) and mortality (33% with RSV vs. 38% without RSV). Instead, a drastic reduction in mortality and in the incidence and duration of VT/VF during the reperfusion phase in animals infused with RSV was observed. Using the highest dose (2.3 × 10^−5^ g/kg), no animal had VF, 20% had VT, and the mortality was 0% [61]. A study of diabetic rats treated with RSV (5 mg/kg/day, intraperitoneally) revealed a decreased frequency and duration of VT/VF and a lowered incidence of other arrhythmia types in the ischaemia–reperfusion model. After 6 min of left coronary artery occlusion and 6 min of reperfusion, there were no rats with VT/VF, and the survival rate was 100% compared to 25.5% VF and 62.5% VT in the control group [62]. Menezes-Rodriguez et al. indicated that in an animal study with 10 min of cardiac ischaemia (occlusion of the left anterior descending coronary artery) and 75 min of recirculation, RSV (1 mg/kg/day, administered orally) significantly reduced the incidence of atrioventricular (AV) block and lethality [63]. Incidence of VAs in the RSV group was 10% lower than in the control group (70% vs. 80%), and mortality was 37.5% lower (25.0% vs. 62.5%). AV block was much less common in the RSV group [63]. In a model with isolated hearts perfused using the Langendorff technique and with 30 min of global ischaemia and 120 min of reperfusion, the RSV also showed a beneficial effect [64]. VF incidence was significantly lower in the RSV group compared to control (median 2.5 vs. 11.5). The incidence of single arrhythmias was reduced from 121 to 36 (median of incidence). CAT, SOD and GPx activity, MDA concentration and nitrite level were significantly higher in the RSV group [64]. Another rat study (an ischaemia–reperfusion model) used dealcoholised Malbec wine (1.1 mg/L RSV) and ethanolic RSV solution (0.5 mL/L) [65]. Heart perfusion with Malbec wine prevented the onset of reperfusion arrhythmias. However, it did not reduce them after the onset, as in the control group. The hearts with arrhythmias that were perfused with RSV recovered sinus rhythm in less than 1 min. Interestingly, the study pointed out that Malbec wine prevented action potential shortening and RSV preserved the resting potential during ischaemia. Overall, arrhythmia in the control group occurred in approximately 90% of cases. In the group with wine and RES, it occurred in about 50% of cases [65]. In rats, RSV significantly increased the doses of aconitine and ouabain required to induce PVCs, VT and VF [66]. It also showed a protective effect on coronary ligation-induced rat arrhythmia and shortened the duration of arrhythmias. Affecting ion channels, RSV decreased ICa current, selectively increased IKs and had no effect on IKr [66]. This action may explain the antiarrhythmic effect.

**Figure 3:**
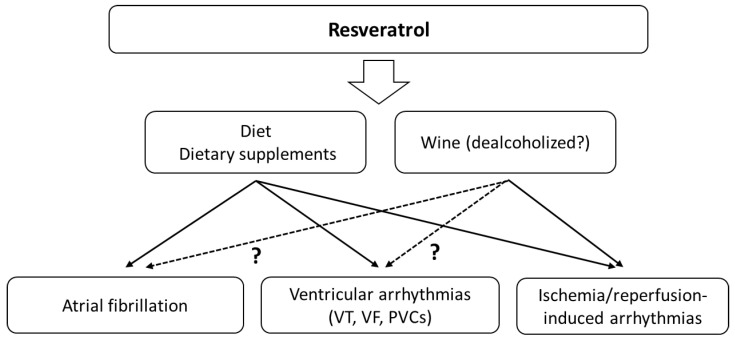
The main effects of resveratrol on cardiac arrhythmias. The effects may also depend on the simultaneous consumption of alcohol.

The above-mentioned studies clearly indicate the possibility of using RSV to treat SVAs, VAs and ischaemia–reperfusion induced arrhythmias (Figure 3). Molecular mechanisms underlying IRI injury in striated muscles involve the production of high levels of ROS. Patients undergoing revascularisation procedures may benefit from reducing the risk of life-threatening arrhythmias or shortening of the arrhythmia in the reperfusion phase.

### 3.5. Statins and Atrial Fibrillation

**Figure 4:**
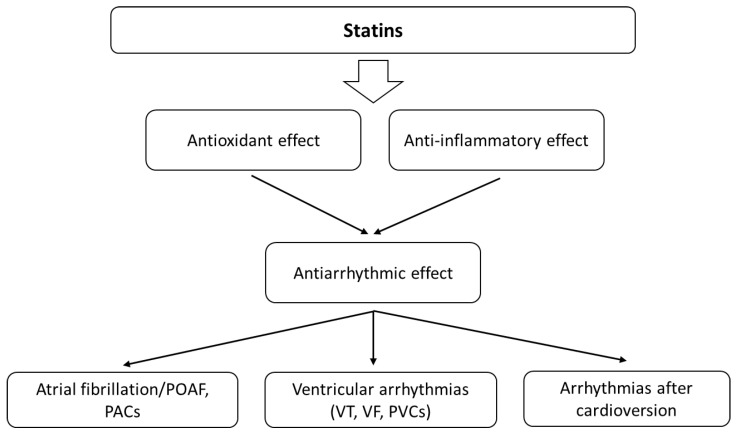
The main effects of statins on cardiac arrhythmias. Their antiarrhythmic activity is probably related to the influence on oxidative stress and the bioavailability of ROS, as well as modulation of the inflammatory response.

Statins are the most commonly prescribed drugs for dyslipidemia and show antioxidant activity. A meta-analysis from 2021 indicates that statin treatment significantly increases the circulating concentrations of GPx and SOD, suggesting an antioxidant effect of these agents [67]. A few randomised trials pose that statin therapy may result in sudden cardiac death (SCD) reduction as an effect of the antiarrhythmic properties of the drugs [68]. Many mechanisms describe the influence of statins on the levels of oxidants and antioxidants [69,70]. They can increase endothelial nitric oxide synthase (eNOS) transcription and the availability of tetrahydrobiopterin (BH4) and eNOS expression through stimulation of protein kinase B (Akt), inhibiting endothelial senescence induced by OS [71,72]. Statins can also affect nicotinamide adenine dinucleotide phosphate oxidases (NADPH) and stimulate antioxidant defences [69]. For example, atorvastatin increased the expression of catalase and manganese superoxide dismutase (MnSOD) [71]. Statins also reduce arrhythmic risk by lowering lipid peroxidation and affect various molecules that play an essential role in both OS and inflammation (Figure 4). The relationship between OS and inflammation is also very strong. OS activates different transcription factors (e.g., NF-κB) that induce the expression of pro-inflammatory cytokines, adhesion molecules and chemokines [73]. Moreover, ROS production is typical of activated inflammatory cells. The direct and indirect associations between the inflammatory process, cellular pathways and arrhythmia are complex. Statins can also reduce oxidized low-density lipoprotein (oxLDL), MDA and thiobarbituric acid reactive substance (TBARS) levels and affect the activity of antioxidant enzymes—SOD, GPx and catalase [74,75,76]. Thus, they can influence the development of arrhythmias.

#### 3.5.1. Animal Studies

Several interesting works have appeared in recent years. One of them describes the antiarrhythmic properties of atorvastatin with regard to AF in an animal model [77]. Animal pretreatment with atorvastatin (10 mg/kg) once a day for 6 weeks had a significantly delayed onset of ouabain-induced arrhythmia in separated atria compared with the control group. Atrial levels of IL-1β, IL-6, and TNF-α were lower in the atorvastatin group [77]. The drug can modulate some inflammatory cytokines but also OS development.

#### 3.5.2. Human Studies

In a study with 64 adult patients undergoing cardiac valve replacement surgery, oral administration of 80 mg of atorvastatin (12 and 2 h preoperatively and on the 2nd, 3rd, 4th and 5th post-operative days) caused a reduction in POAF incidence [78]. In addition, the patients showed a less intense inflammatory process. It also discussed associations between the development of POAF, the inflammatory process and OS. Similar data were obtained earlier in an analysis of 12 randomised controlled trials. Statin therapy was associated with a decrease in the risk of post-operative AF [79]. Furthermore, in another study with 199 patients, perioperative atorvastatin treatment prevented the myocardial nitroso–redox imbalance, and the therapy caused an increase in atrial superoxide production and reduction in NOS activity [80]. In an experiment with atrial samples from 130 patients undergoing cardiac surgery, atorvastatin caused inhibition of Rac1 (Ras-related C3 botulinum toxin substrate 1) and NOX2 (NADPH oxidase) activity in right atrial samples from patients who developed post-operative AF [81]. Upregulation of atrial NADPH oxidases is a natural event in the development of AF and may explain the effect of statins in preventing arrhythmia. It should be noted that actual findings suggest that statin treatment is ineffective in the primary prevention of AF [82]. On the other hand, it can positively affect secondary prevention. Given the role of redox status in AF, statins may be helpful in the reduction in AF recurrence rates after successful cardioversion. A new single-centre registry study from 2021, including 454 patients, showed that statin therapy resulted in an absolute risk reduction of 27.5% for recurrent AF after cardioversion [83]. The study took into account the pleiotropic effect of statins, likely resulting in a decreased OS and anti-inflammatory effect. In the light of the results obtained so far, it seems that the combination of statin treatment with vitamins C and E supplementation may be effective in treating AF, including POAF.

### 3.6. Statins and Ventricular Arrhythmias

#### 3.6.1. Animal Studies

Unfortunately, there is a lack of research on the antioxidant effect of statins in VAs. One in vitro study analysed the effect of oxLDL on intracellular Ca^2+^ handling in adult rat ventricular cardiomyocytes [84]. It revealed that oxLDL could cause SR-Ca^2+^ leak and spontaneous Ca^2+^ sparks and could induce a pro-arrhythmogenic profile. The same study also showed that people with well-controlled stable coronary artery disease, usually treated with statins, had significantly lower oxLDL levels than subjects at high cardiovascular risk [84]. This indicates that the antioxidant properties of statins may be useful in treating arrhythmias. Ma et al. [85] reported similar results, demonstrating that atorvastatin could inhibit the ICa,L current via suppressing ROS production in neonatal rat ventricular myocytes.

#### 3.6.2. Human Studies

Some studies demonstrate that statins decrease VAs in ICD patients. A very interesting study of 304 subjects with ICD indicated that patients on statins had a significantly lower level of derivatives of ROS (DROMs) (a measure of lipid peroxides), and the DROMs and statins were strongly associated with ICD events [86]. Importantly, DROMs were the dominant predictor of the ICD event rate [86]. This indicates that statins exert an antiarrhythmic effect by influencing the oxidative status. Exciting research was published in 2016 by Chen et al. regarding the effects of statin on arrhythmia in young, healthy persons with sleep deprivation [87]. Seventy-two young, healthy participants were recruited from the army and remained awake in the sleep laboratory for 48 h. Participants were randomised to statin or placebo groups. Patients in the statin group were given 20 mg of atorvastatin (daily) 3 days before sleep deprivation and for 2 days during sleep deprivation. The study assessed, inter alia, the activity or concentration of OS parameters (SOD and MDA) and frequency of arrhythmia—premature atrial complexes (PACs) and PVCs—simultaneously. After 48 h of sleep deprivation, the frequency of PACs and PVCs was significantly decreased in the statin group (seven PACs/hour, and five PVCs/hour vs. fourteen PACs and nine PVCs in the control group). MDA concentration was 2.51 nmol/mL lower in the statin group. SOD activity was also lower, but without statistical significance. The study concluded that a possible mechanism of the influence of statins on arrhythmias is related to OS [87]. There is a relationship between redox status and the antioxidant effect of statins, not only in life-threatening ventricular arrhythmias but also in mild ones, including PVCs.

### 3.7. Other Studies with Antioxidants

In addition to the OS modulators mentioned above, other antioxidants have also been investigated. For instance, it was shown that mitochondrial OS plays a central role in gap junction remodelling and arrhythmia [88]. An animal model of treatment with MitoTEMPO ((2-(2,2,6,6-tetramethylpiperidin-1-oxyl-4-ylamino)-2-oxoethyl)triphenylphosphonium chloride, a specific scavenger of mitochondrial superoxide) revealed decreased spontaneous PVCs and VT inducibility and reduced SCD by reducing mitochondrial ROS levels. MitoTEMPO treatment also increased the Cx-43 level at the gap junctions to 62% of the control value and increased the gap junction conduction [88]. Cx43 is the major structural protein of ventricular gap junctions. A significant decrease in Cx43 expression causes sudden arrhythmic death [89]. In a guinea pig model, MitoTEMPO eliminated SCD by decreasing dispersion of repolarisation (indexed by the QT variability index) and VAs [9]. Moreover, treatment with MitoTEMPO preserved cardiac function and prevented the onset of HF [9]. A model of I/R-induced arrhythmia in cats demonstrated that injections of NOS inhibitor—L-NAME (NG-nitro-L-arginine methyl ester)—decreased the incidence of arrhythmias by 40% and eliminated reperfusion-induced VT/VF [90]. L-NAME can also counteract the proarrhythmic effects of carbon monoxide [91]. Unfortunately, there are not many new studies on NOS inhibitors, and the data on L-NAME are inconclusive. Some studies indicate a possible proarrhythmic effect of L-NAME. Inhibition of NO increases VF in isolated rat hearts subjected to I/R [92]. MitoTEMPO and L-NAME are not used in clinical practice (especially MitoTEMPO), and most results come from animal model studies. Although it is traditionally not classified as an antioxidant, L-NAME affects NO synthesis and redox balance. Magnolol, an active component of Magnolia officinalis is about 1000-times more potent than α-tocopherol in inhibiting lipid peroxidation. It was shown in 1996 that magnolol significantly reduced the incidence and duration of I/R-induced VT/VF in rats during 30 min of coronary ligation and 10 min of reperfusion [93]. A similar effect was observed using honokiol (polyphenolic compound, similar to magnolol traditionally used in medical practice) [94]. Other antioxidants, found in pomegranates (*Punica granatum* L.), may cause a significant reduction in the occurrence of arrhythmias [95]. Rat hearts perfused with Krebs solution with pomegranate lyophilised juice (1.8 mg/mL, 2% *v*/*v*) had a lower incidence and shorter duration of VF during ischaemia and reperfusion phases. Single salvo VT was significantly lower in the pomegranate group compared to the control. Antioxidant enzymes, such as SOD, GPx and catalase, demonstrated a significant increase in the group with pomegranate juice. The addition of L-NAME reduced these effects [95]. Arctigenin (a natural lignan compound extracted from Arctium lappa) also shows antiarrhythmic activity. In an I/R injured rat heart model, arctigenin significantly reduced the incidence and duration of VT, VF and ventricular ectopic beats [96]. Rats treated with arctigenin had no VF during reperfusion and markedly enhanced activities of SOD and GPx, as well as reduced MDA levels. Administration of arctigenin (50 mg/kg/day and 200 mg/kg/ day) significantly increased the expression of Nrf2 (nuclear factor erythroid 2-related factor 2), Trx1 (thioredoxin-1) and Nox1 (NADPH Oxidase 1) [96]. Interesting research also concerns melatonin, which appears as a key molecule in heart ageing. Melatonin was shown to have an antiarrhythmic effect through a decrease in OS, the stimulation of antioxidant enzymes [97] and a decrease in fibrosis and apoptosis associated with AT1 (angiotensin 1) reduction and HSP70-VDR (heat shock protein 70—vitamin D receptor) increase [98]. Melatonin cardioprotection against OS and arrhythmias could also be explained by modulation of ion channels and Cx43 expression [99]. A detailed description of the molecular mechanisms can be found in Segovia-Roldan et al. [100]. One of the latest studies from 2021 demonstrates that melatonin protects against the development of early VF in the myocardial infarction animal model [101]. A randomised controlled trial indicated that melatonin might help modulate OS and the duration of AF following CABG surgery [102]. The feasibility of using antioxidative effects was also noticed in ICD patients, who form a specific group. It often includes people with HF who avoid physical exercises and have increased OS parameters. Some observations indicate that patients with chronic HF who also had ICDs or cardiac resynchronisation therapy-defibrillators (CRT-D), and who performed different stretching exercises (30 s per stretching exercise on a floor mat or chair, followed by 20 **s** of relaxation, with each exercise being performed twice and with all 7 stretches amounting to 20 min in total), had significantly decreased ROS and malondialdehyde-modified low-density lipoprotein cholesterol (MDA-LDL) level [103]. Exercise causes the increase in intracellular antioxidants in skeletal muscles and attenuation of oxidative stress. During the study, patients had no ICD shocks. This may indicate a positive role of antioxidants in this group of patients.

## 4. Conclusions

**Figure 5:**
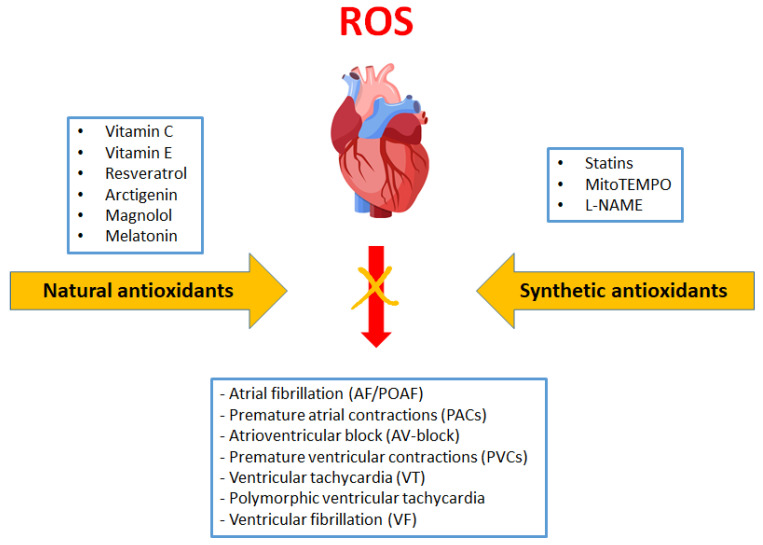
Natural and synthetic antioxidants in the proposed therapy for cardiac arrhythmias.

Most of the available studies show a significant relationship between the availability of antioxidants, redox status and OS and cardiac arrhythmias (Table 1). Many cellular and molecular mechanisms responsible for developing arrhythmias during OS have been described. For example, OS plays a key role in the development of AF. It may also contribute to life-threatening arrhythmias—VT and VF. However, clinical studies on ventricular arrhythmias and OS are lacking. At the moment, attention is paid chiefly to the role of antioxidants in AF and POAF treatment. Attempts to use antioxidants as adjuvant drugs to treat cardiac arrhythmias are justified, because routinely used antiarrhythmic medications have many potential interactions and side effects. Paradoxically, antiarrhythmic drugs may also be proarrhythmic, that is, they may increase existing arrhythmia or induce arrhythmia. The use of various antioxidants, including vitamins and polyphenols, is likely to improve standard treatment effects (Figure 5). Combination therapy could potentially reduce the doses of standard drugs. Natural antioxidants are safe and have limited side effects, and research suggests they are associated with a low risk for the patient. Scavenging ROS production or downregulating enzymes related to ROS could be investigated as novel therapeutic approaches. The studies conducted so far indicate that their application may play a significant role in preventing and treating cardiac arrhythmias. Antioxidants could potentially be used not only as direct ROS scavengers but also in protecting against cardiac fibrosis and contributing to maintaining the normal function of the heart’s conduction system. Most available research is based on animal and cellular models. There is a great need for clinical trials in humans. In our opinion, antioxidants can be useful not only in patients with chronic arrhythmias but also in emergency and critically ill patients, where redox imbalances are very pronounced and constitute a pathomechanism of many diseases. Future research should focus on the development of specific antioxidant drugs, and even now, certain natural compounds (vitamins C and E, resveratrol, polyphenols etc.) can serve as supportive and safe therapies.

## 5. Limitations of the Data and Their Interpretation

Currently, little is known about ROS-involved mechanisms inducing arrhythmia. The studies published so far have many limitations. Many of them were conducted only on animals. The data do not provide robust evidence that the antioxidants induce direct effects influencing the development of arrhythmias. Thorough research is needed to assess “cause” and “effect”. There are also some conflicting data showing failure of antioxidant interventions in the treatment of arrhythmias. On the basis of inconsistent data from both experimental and clinical studies, it is difficult to demonstrate a clearly positive therapeutic effect of antioxidants. The action of ROS and antioxidants can be multidirectional. For example, a very important signal molecule—H_2_O_2_—can cause arrhythmia by increasing the late sodium current, but at the same time can reduce SCN5A transcription and inhibit total sodium current. This condition can lead to ROS-mediated EAD-related arrhythmias, but can also provide a substrate for re-entry. Mitochondrial antioxidants may be useful in treating these arrhythmias, but it is necessary to take into account their possible negative systemic effects. It is not known whether natural antioxidants have the same effect. It seems that the form of their administration is also important. Antioxidants can have a positive effect, but combining them, for example, with alcohol (wine) can sometimes exacerbate arrhythmia. Moreover, not all results can be interpreted in terms of OS. Changes in the activity of antioxidant enzymes and an increase in the concentration of reactive oxygen species or oxidation products do not always indicate the occurrence of OS.

## Figures and Tables

**Table 1:** Summary of selected studies in animal models and clinical trials.

Study	Used Antioxidant/Drug	Study Model	End-Point	Primary Findings
Frolkis et al. [36]	Vitamin E	Animal	SVA	Animals treated with vitamin E had lower incidence rate of supraventricular arrhythmias
Korantzopoulos et al. [37]	Vitamin C	Human	AF	Vitamin C reduces recurrence rates of AF after electrical cardioversion of AF
Rodrigo et al. [38]	Vitamin C, E, PUFAs	Human	POAF	Antioxidants significantly reduce the risk of POAFs
Mirhoseini et al. [41]	Vitamin C, E, selenium	Human	Arrhythmias in critically ill trauma patients	The use of antioxidants was associated with a longer expected survival time but did not decrease the incidence of atrial arrhythmias
Violi et al. [44]	Vitamin C, E, PUFAs	Human	POAF	13% reduction in POAF after CABG
Guo et al. [45]	Vitamin C, E, PUFAs	Human	POAF	PUFAs without antioxidants did not reduce the incidence of POAF but combination therapy with vitamins C and E reduced the incidence of POAF by 68%
Sethi et al. [46]	Vitamin E	Animal	VT	Vitamin E reduced the incidence of VT by approx. 50%
Sethi et al. [47]	Vitamin E	Animal	Arrhythmia after ischemia–reperfusion	Decrease in PVCs 21 days after coronary artery occlusion
Karahaliou et al. [48]	Vitamin C, deferoxamine	Animal	VT/VF	Over 60% risk reduction of VT/VF in reperfusion phase after ischemia
Chen et al. [49]	Vitamin E	Animal	QT interval prolongation	Treatment with vitamin E shortens QTc interval
Barbosa et al. [50]	Vitamin C, E	Human	Arrhythmia in patients with Chagas disease	Significant reduction in PVCs
Chong et al. [55]	Resveratrol	Animal	AF	Reduction in AF and incidence of triggered activity
Zhang et al. [56]	Resveratrol	Animal	AF	Reduction in inducibility and duration of AF
Frommeyer et al. [59]	Resveratrol, piceatannol	Animal	AF	Significant reduction in AF incidence
Hung et al. [61]	Resveratrol	Animal	VT/VF	Reduction in the incidence and duration of VT/VF during reperfusion phase
Kaya et al. [62]	Resveratrol	Animal	VT/VF	Reduction in frequency and duration of VT/VF during reperfusion phase
Rodriguez et al. [63]	Resveratrol	Animal	Ventricular arrhythmia	Reduction in the incidence of atrioventricular block, lethality and VAs
Kazemirad et al. [64]	Resveratrol	Animal	VF	Lower incidence of VF and single arrhythmias
Prado et al. [65]	Resveratrol (wine)	Animal	Arrhythmias after ischemia–reperfusion	Prevention of arrhythmias in the reperfusion phase, faster recovery of sinus rhythm
Najjari et al. [77]	Atorwastatin	Animal	AF	Delayed time of onset of ouabaine-induced atrial arrhythmia
Allah et al. [78]	Atorwastatin	Human	POAF	Reduction in POAF incidence
Fiedler et al. [83]	Atorwastatin	Human	AF	Reduction in AF recurrence rate after successful cardioversion
Bloom et al. [86]	Various statins	Human	VAs, ICD events	Use of statins is associated with reduced DROMs and fewer ICD events
Chen et al. [87]	Atorwastatin	Human	SVA, VAs	Significantly decreased PACs and PVCs frequency
Sovari et al. [88]	MitoTEMPO	Animal	PVCs, VT, SCD	Decreased spontaneous PVCs, decreased VT inducibility and reduction SCD
Dey et al. [9]	MitoTEMPO	Animal	SCD	Decreased risk of VAs and SCD
Kukushkina et al. [90]	L-NAME	Animal	Arrhythmia after ischemia–reperfusion	Decreased incidence of ventricular arrhythmias and eliminated of reperfusion-induced VT/VF
Pabla et al. [92]	L-NAME	Animal	VF	L-NAME had no significant effect on the incidence of ischemia-induced VF, but increased the incidence of reperfusion-induced VF
Hong et al. [93]	Magnolol	Animal	VT/VF	Significantly reduced incidence and duration of I/R-induced VT/VF
Kazemirad et al. [95]	*Punica granatum* L. polyphenols	Animal	VF	Lower incidence and reduced duration of VF during ischemia and reperfusion phase
Yang et al. [96]	Arctigenin	Animal	VT/VF	Significantly reduced incidence and duration of VT/VF and ventricular ectopic beats

## Data Availability

Not applicable.
